# Microbial–Clay Mineral Interactions: Mechanisms and Applications in Environmental Remediation

**DOI:** 10.3390/toxics14100900

**Published:** 2026-10-11

**Authors:** Xinlin Huang, Qiuyi Li, Mi Feng, Chongmin Liu, Haoyu Shi, Dunqiu Wang

**Affiliations:** 1 College of Environmental Science and Engineering, Guilin University of Technology, Guilin 541004, China; xini197441@163.com (X.H.); lqy1626743718@163.com (Q.L.); 18956191869@163.com (H.S.); wangdunqiu@sohu.com (D.W.); 2 Guangxi Key Laboratory of Environmental Pollution Control Theory and Technology, Guilin University of Technology, Guilin 541006, China; 3 University Engineering Research Center of Watershed Protection and Green Development, Guangxi, Guilin University of Technology, Guilin 541006, China; 4 Key Laboratory of Carbon Emission and Pollutant Collaborative Control, Education Department of Guangxi Zhuang Autonomous Region, Guilin University of Technology, Guilin 541006, China

**Keywords:** microorganisms, clay minerals, interactions, co-evolution, biomineralization

## Abstract

Microbial–clay mineral interactions play a pivotal role in environmental remediation, biogeochemical cycling, and the long-term evolution of the Earth’s systems. This review critically synthesizes the fundamental mechanisms, reactive pathways, and environmental implications of microbial–clay mineral interactions. Clay minerals, owing to their large specific surface area, high cation exchange capacity, and tunable surface charge, are shown to sustain microbial metabolism by offering physical shelter and serving as sources of essential nutrients. Concurrently, microorganisms mediate the dissolution, structural alteration, and neoformation of clay phases via proton production, electron transfer reactions, and genetic regulatory networks, thereby establishing a dynamic synergistic relationship. In environmental remediation, clay–microorganism complexes have been demonstrated to promote heavy metal stabilization. They concurrently expedite the breakdown of organic contaminants, including polycyclic aromatic hydrocarbons (PAHs). Nevertheless, major knowledge gaps persist, especially concerning the multiscale complexity of interfacial behaviors and still-elusive molecular-scale interaction mechanisms. Finally, several priority directions for the future are proposed, including the integration of metagenomics and molecular modeling, the design of biomarker-based monitoring tools, and the scalable translation of bench-scale discoveries and laboratory insights into field-scale remediation technologies. Collectively, this work advances fundamental knowledge of key microbe–mineral biogeochemical processes and provides a scientific basis for the development of sustainable, nature-based environmental remediation strategies.

## 1. Introduction

Microorganisms constitute the cornerstone of Earth’s ecosystems and are critically involved in the transformation of environmental pollutants [1,2]. Through diverse metabolic pathways, they can efficiently degrade organic contaminants. In parallel, heavy metals are subject to immobilization or valence-state alteration via biosorption, precipitation, redox reactions, and biomineralization [3,4]. These fundamental microbial processes underpin environmental self-purification and ecological restoration. Meanwhile, clay minerals, as ubiquitous reactive constituents in natural environments, possess a unique layered structure, high specific surface area, and exceptional ion exchange capacities [5,6]. These intrinsic attributes render them critical adsorbents and reactive interfaces that significantly modulate the migration and transformation of contaminants.

When microbes and clay minerals coexist in the environment, they do not function in isolation but rather establish a dynamic and interdependent partnership. This synergistic interaction serves as a fundamental driver of material cycling in the Earth’s critical zone [3,7], forming a complex “mineral-microbe-environment” feedback system [8]. Within this system, clay minerals act as microbial habitats, offering physical shelter, essential nutrients, and reactive sites to sustain their survival and activity [9]. In turn, microbial activity fundamentally transforms and reshapes the structure, morphology, and surface chemistry of clay minerals through metabolite secretion, redox reactions, and mediating biomineralization processes. This mutual reinforcement enhances both heavy-metal immobilization and organic-pollutant degradation. In clay–microbe complexes, heavy metals can be stabilized through additional adsorption sites, biofilm formation, and changes in metal valence. Meanwhile, mineral surfaces can concentrate organic pollutants and support microbial growth, thereby promoting coupled adsorption and biodegradation processes [10,11,12].

**Figure 1:**
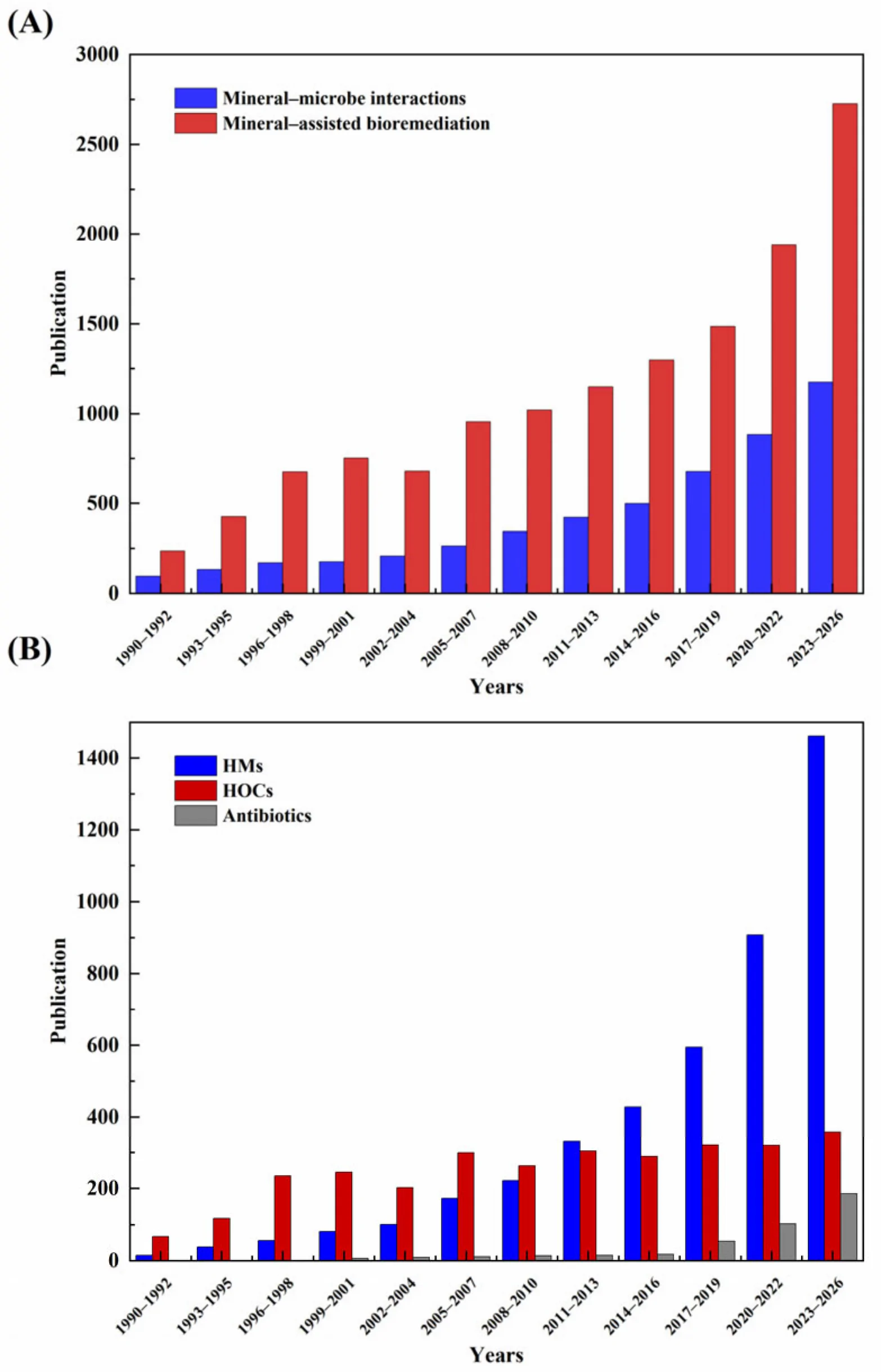
Bibliometric analysis of research trends in clay mineral–microbe interactions and mineral-assisted bioremediation from 1990 to 2026. (**A**) Annual publications on clay mineral–microbe interactions and mineral-assisted bioremediation. (**B**) Annual publications related to mineral-assisted bioremediation of heavy metals, hydrophobic organic contaminants, and antibiotics. (Figure 1 was generated using Origin).

Over recent decades, substantial progress has been achieved in understanding microbe-clay mineral interactions, largely driven by the integration of interdisciplinary approaches spanning molecular biology, spectroscopy, and geochemistry. The number of relevant publications has risen steadily since 1990, with particularly strong growth in the past five years (Figure 1A). Notably, recent investigations have increasingly emphasized mineral-mediated biotransformation and bioremediation, particularly for heavy metals, organic contaminants, and antibiotics (Figure 1B). This trend highlights the growing recognition of the critical role that microbe–mineral interfacial processes play in environmental remediation. Earlier studies have mainly concentrated on (i) fundamental microbe-clay mineral interactions, (ii) mineral-assisted biotransformation and biodegradation mechanisms, and (iii) the remediation of typical hazardous pollutants, particularly heavy metals, organic contaminants, and antibiotics.

In recent years, rapid advancements in high-resolution microscopic imaging, synchrotron-based spectroscopy, and molecular biological approaches have propelled research on clay mineral–microbe interactions beyond early phenomenological descriptions toward a mechanistic understanding of interfacial processes. Accumulating evidence indicates that clay minerals are not merely passive carriers; rather, their layered structures, surface reactive sites, exchangeable cations, and interlayer microenvironments can strongly regulate microbial adhesion, metabolic responses, and elemental cycling. Conversely, microorganisms can actively reshape mineral surfaces and reactivity through extracellular polymeric substances, ligand-promoted dissolution, and redox-driven transformation, thereby establishing a dynamic and reciprocal mineral–microbe interfacial system [3,13,14]. Among diverse clay mineral types, montmorillonite, as a typical 2:1 phyllosilicate, generally exhibits high specific surface area, strong cation-exchange capacity, and expandable interlayers, all of which favor pollutant enrichment and interfacial reactions. In contrast, kaolinite, a representative 1:1 clay mineral, is commonly used as a model substrate for investigating microbial adhesion and mineral surface heterogeneity [3,14]. At the mechanistic level, current research predominantly focuses on three interconnected pathways: physical protection afforded by layered and interlayer domains, chemical regulation of contaminant adsorption and transformation at mineral surfaces, and biomineralization processes that affect elemental immobilization and long-term stability [14]. In addition, microbe-clay mineral composite systems are increasingly explored for remediating heavy metals, hydrophobic organic contaminants, and antibiotics [14]. For example, during phenanthrene biodegradation, the combined system of *Sphingomonas* sp. GY2B and modified montmorillonite has been shown to alter the surface morphology, structural order, and elemental release behavior of montmorillonite, highlighting that clay minerals function not merely as adsorptive supports but also as active reaction components participating in and reciprocally influencing pollutant biotransformation [15].

However, this escalating research focus has not yet translated into a comprehensive understanding of the intricate interactions between microorganisms and clay minerals. A principal knowledge gap lies in the fragmented perception of mechanisms and their interconnections. Much of the existing literature addresses isolated mechanisms (e.g., physical protection, chemical regulation, biomineralization) without integrating them to uncover potential synergies or antagonisms [9,16,17]. A telling example is extracellular polymeric substance (EPS)-mediated biofilm formation on montmorillonite, which provides physical protection and concurrently facilitates chemical adsorption of heavy metals via its functional groups [18,19]. Yet, the quantitative influence of mineralogy on biofilm architecture—and consequently on the balance between physical shielding efficiency and chemical adsorption capacity—remains unresolved. The field is further impeded by a ‘translation gap’ that separates fundamental research from practical application. Molecular-level insights have yet to be effectively converted into innovative remediation strategies [16,20]. Critical questions remain unanswered: how to design high-performance engineered bio-mineral composites guided by genetic principles, or how to achieve the precise manipulation of carbon sequestration and metal immobilization in the field [21,22]. Moreover, the behavior of emerging pollutants (e.g., antibiotics, microplastics) within these systems remains largely unknown [23]. This divide between fundamental knowledge and practical implementation fundamentally constrains the deployment of microbe-mineral interactions for addressing real-world environmental challenges.

Accordingly, this review aims to bridge these gaps by developing an integrated framework that connects molecular mechanisms to engineering applications, thereby systematically synthesizing the principles and applications of mineral–microbe interactions. The article first introduces the major clay mineral types and representative microorganisms (Section 2). It then examines the principal mechanisms of clay mineral–microorganism interactions, including physical and biochemical regulation, chemical regulation, and biomineralization (Section 3). Section 4 discusses their applications in heavy-metal immobilization and organic-pollutant remediation. Finally, the review discusses strategies for integrating microscopic mechanistic understanding with macroscopic ecosystem management, aiming to provide a theoretical basis and future directions for the development of efficient and stable in situ bioremediation technologies.

### Literature Search and Selection Strategy

A literature search was conducted using the Web of Science Core Collection to characterize publication trends related to clay mineral–microorganism interactions and mineral-assisted bioremediation. The search was performed on 20 August 2026, covering the period from 1990 to 2026. The Topic field (TS) was used for all searches, and only English-language Articles and Review Articles indexed in the Science Citation Index Expanded (SCI-EXPANDED) were included.

For Figure 1A, two independent search datasets were constructed. The first dataset focused specifically on clay mineral–microorganism interactions and combined terms describing representative clay minerals (e.g., montmorillonite, kaolinite, illite, smectite, vermiculite, and chlorite), microorganisms, and interaction processes such as adhesion, colonization, transformation, and weathering. The second dataset represented the broader field of mineral-assisted bioremediation and combined mineral-related terms, including both clay minerals and environmentally relevant Fe-bearing minerals, with microbial and remediation-related terms. This broader dataset was used to provide a contextual comparison with the more specific literature on clay mineral–microorganism interactions.

For Figure 1B, three independent pollutant-specific datasets were generated using the mineral-assisted bioremediation search framework. A common combination of mineral-related, microbial-related, and remediation-related terms was further combined with pollutant-specific keywords representing (i) heavy metals and metalloids, (ii) hydrophobic organic contaminants (HOCs), and (iii) antibiotics. The heavy-metal dataset included representative contaminants such as Cd, Pb, Cr, As, Hg, Cu, Zn, and Ni; the HOC dataset included polycyclic aromatic hydrocarbons, petroleum hydrocarbons, chlorinated hydrocarbons, and polychlorinated biphenyls; and the antibiotic dataset included major antibiotic classes and representative compounds such as tetracyclines, sulfonamides, fluoroquinolones, macrolides, chloramphenicol, and amoxicillin.

For the bibliometric analysis, records meeting the predefined database, document-type, language, publication-period, and search-query criteria were included in the corresponding dataset. Annual publication counts were recorded separately for each search dataset and used to construct Figure 1. The complete Boolean search strings and search conditions for all five datasets are provided in Table S1.

## 2. Fundamental Components

### 2.1. Clay Minerals

**Figure 2:**
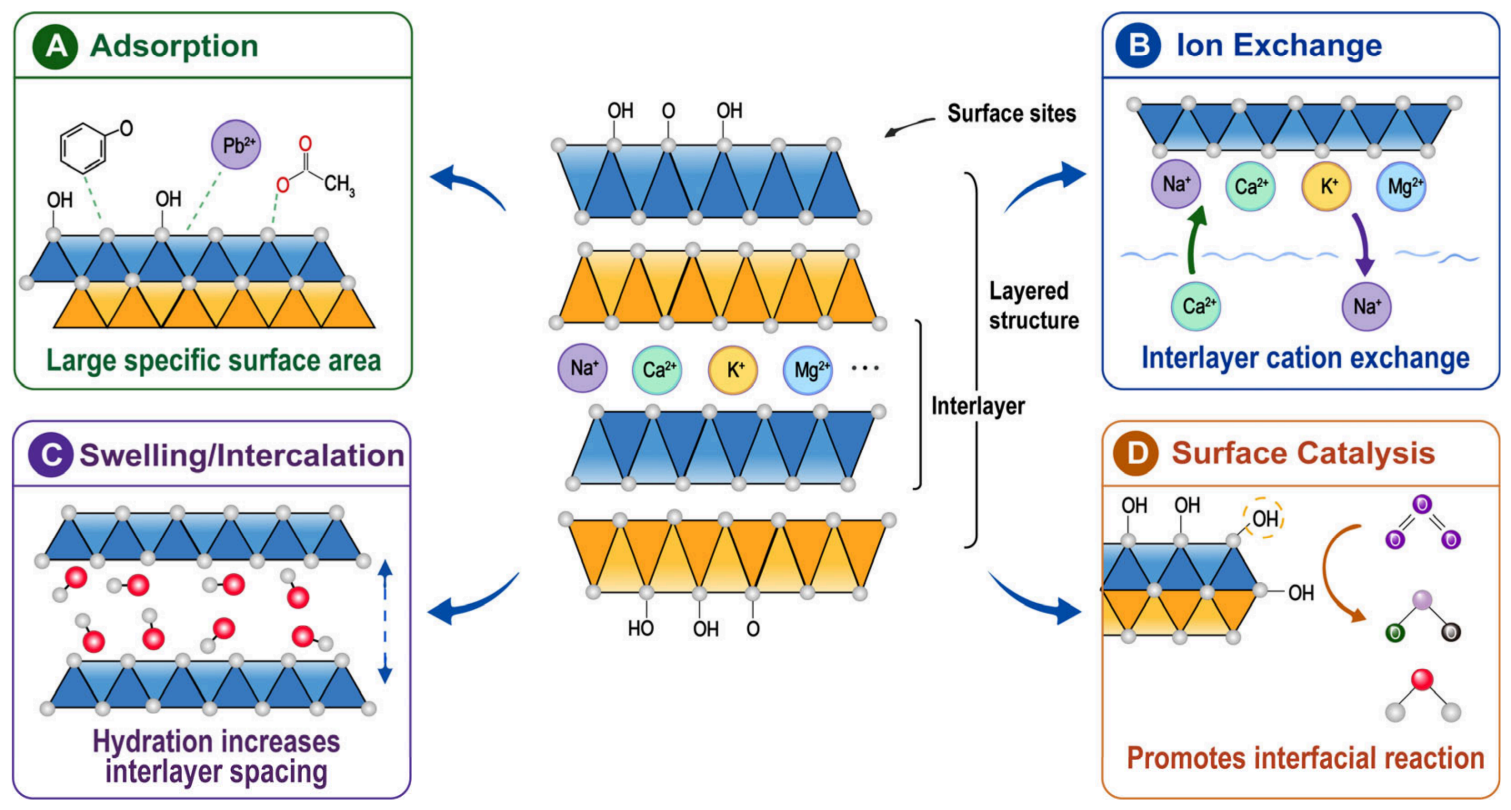
Structural characteristics and functional properties of clay minerals: (**A**) adsorption; (**B**) ion exchange; (**C**) swelling/intercalation; (**D**) surface catalysis. Figure 2, Figure 3, Figure 4, Figure 5, Figure 6, Figure 7, Figure 8 and Figure 9 were created by the authors using Adobe Illustrator.

Clay minerals constitute a class of naturally occurring, fine-grained hydrous aluminosilicates, typically with particle sizes below 2 micrometers [24]. They originate primarily from the chemical weathering of silicate-based rocks and exhibit a layered architecture composed of silicon-oxygen tetrahedral sheets and aluminum-oxygen (or magnesium/iron-oxygen) octahedral sheets. The arrangement and stacking sequence of these sheets underpin classification into several structural groups, including 1:1 type (e.g., kaolinite, consisting of a tetrahedral and one octahedral sheet), 2:1 type (e.g., montmorillonite, featuring an octahedral sheet sandwiched between two tetrahedral sheets), and 2:1:1 type (e.g., chlorite, which incorporates an additional octahedral layer) [25]. The distinctive physicochemical properties of clay minerals arise from their layered configuration and associated surface chemistry. The layered structure, surface hydroxyl groups, and exchangeable interlayer cations provide multiple reactive sites for pollutant interactions (Figure 2). Their large specific surface area facilitates the adsorption of organic and inorganic contaminants, while negatively charged layers enable cation exchange with metal ions [5,6]. Expandable clay minerals such as montmorillonite can also accommodate water molecules and organic compounds within their interlayers through swelling and intercalation processes [25]. In addition, structural defects and reactive surface sites may participate in catalytic reactions and promote interfacial transformation processes [5]. These properties collectively underpin the functional versatility of clay minerals in environmental systems. For example, the pronounced swelling characteristic of montmorillonite is utilized in environmental remediation and nanocomposite synthesis [25]. In comparison, non-swelling clay minerals such as illite and kaolinite provide considerable cation exchange capability and structural stability, making them valuable in areas such as soil enhancement, ceramic production, and animal nutrition [26]. Due to their adsorption and catalytic properties, clay minerals also serve as key participants in biogeochemical cycles and are widely utilized as functional materials [27]. Their structural configuration influences not only mechanical and physical behaviors but also interactions with water and organic compounds—attributes that are essential in environmental and materials engineering [5,27]. As an example, when employed as supplementary cementitious materials, the specific mineralogy of clays (e.g., the relative abundance of kaolinite, illite, or montmorillonite as shown in Table 1) can significantly affect the rheological and durability performance of the resulting construction materials [28].

Overall, the defining features of clay minerals are their layered aluminosilicate composition and variable chemical makeup, which collectively give rise to a wide range of functional properties. Their classification is systematically based on the stacking pattern and the ratio of tetrahedral to octahedral sheets [29]. These inherent characteristics enable their critical roles in natural systems—such as pedogenesis and nutrient cycling—as well as their extensive utility in industrial and environmental applications [5,24,25,27,29]. These structural properties also provide the physicochemical basis for subsequent interactions between clay minerals and microorganisms, including microbial adhesion, nutrient exchange, and mineral-mediated transformation processes [3,14].

**Table 1:** Comparison of key properties of kaolinite, montmorillonite, and illite.

Clay Mineral	Specific Surface Area (m^2^/g)			Cation Exchange Capacity (CEC)	Structural Features	Citations
	Internal Surface Area	External Area	Total Surface Area			
Kaolinite	0–5	10–25	10–30	Low (3–15 meq/100 g)	1:1 layer, non-expanding, low charge	[30,31,32]
Montmorillonite	600–800	40–100	640–900	High (80–150 meq/100 g)	2:1 layer, expanding, high charge	[30,31,32]
Illite	0–10	15–35	15–45	Moderate (20–40 meq/100 g)	2:1 layer, non-expanding, K^+^ in interlayer	[30,31,32]

### 2.2. Types of Microorganisms

Clay minerals are widely distributed in soils, sediments, and aquatic environments, where they interact with diverse microorganisms and form dynamic mineral–microbe interfaces. These interactions regulate microbial colonization, mineral transformation, elemental cycling, and environmental pollutant behavior.

The interactions between clay minerals and microorganisms involve a diverse array of microbial taxa, which form complex synergistic relationships through their distinct physiological traits and metabolic activities. Major microbial groups engaged in these processes include bacteria, fungi, archaea, and algae.

**Figure 3:**
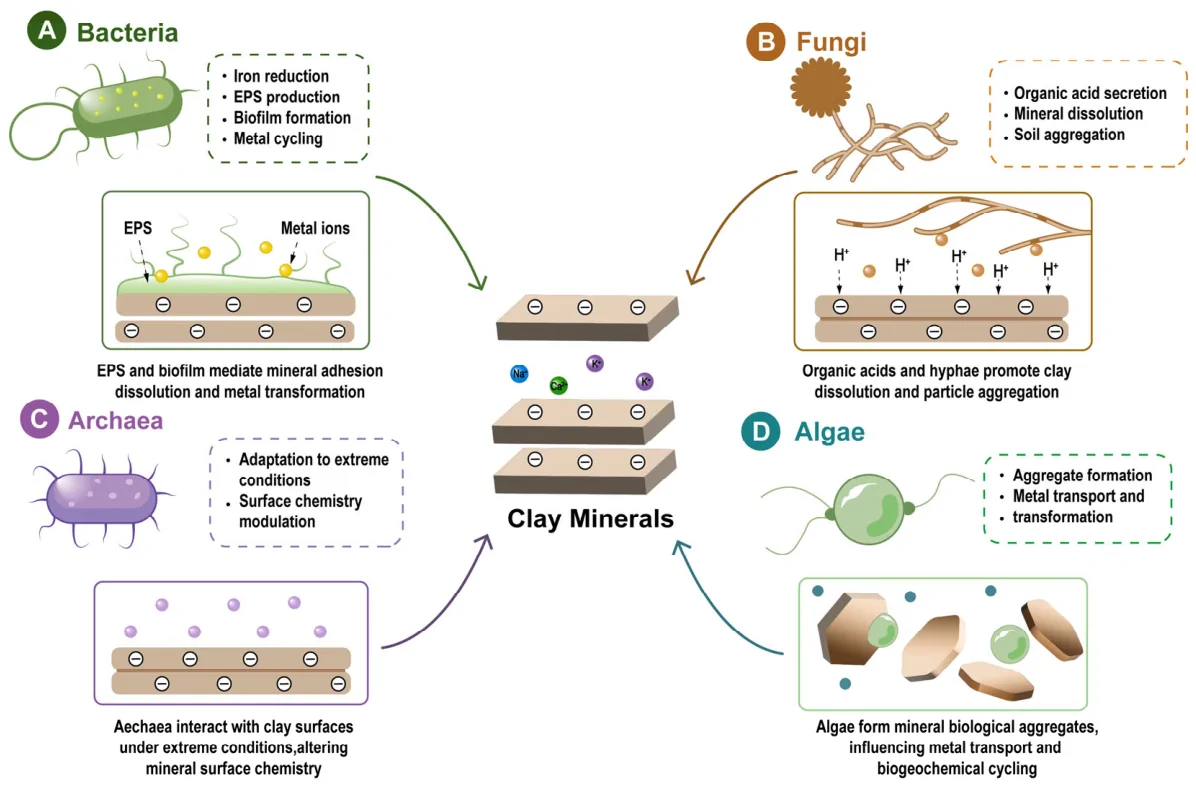
Functional interactions between clay minerals and microorganisms in environmental systems: (**A**) bacteria; (**B**) fungi; (**C**) archaea; (**D**) algae.

Different microbial groups interact with clay minerals through distinct pathways, resulting in diverse ecological functions and mineral transformation processes (Figure 3). Among these microorganisms, bacteria represent the most extensively studied group in clay–microbe systems. They attach to mineral surfaces through electrostatic interactions, surface proteins, and EPS, which promote biofilm formation and establish mineral-associated microbial communities. These interactions can further regulate mineral dissolution, redox reactions, and elemental cycling.

Fungi mainly interact with clay minerals through hyphal attachment and organic acid secretion. Their filamentous growth and metabolic activities promote mineral dissolution, element release, and particle aggregation, thereby modifying mineral structures and creating heterogeneous microhabitats [3,33,34]. In contrast, archaea, particularly extremophilic species, contribute to mineral transformation and geochemical cycling under extreme environmental conditions, such as high salinity, temperature, or pH [3]. Algae interact with clay particles mainly through extracellular polymers and mineral aggregation processes, influencing the transport and transformation of associated elements in aquatic environments [35].

Microbial groups exhibit distinct but complementary interaction strategies with clay minerals. These interactions determine microbial colonization patterns, mineral reactivity, and the subsequent environmental functions of clay–microbe systems. The representative microorganisms and their ecological roles are summarized in Table 2. Both Gram-negative (e.g., *Escherichia coli*, *Pseudomonas*, *Shewanella*) and Gram-positive (e.g., *Bacillus*, *Tessaracoccus*, *Flaviflexus*, *Halolactibacillus*, *Alkaliphilus*, *Clostridium*) bacteria have been documented to participate in various interaction pathways [36,37,38]. Certain species, including *Shewanella* and *Geobacter*, are capable of reducing structural Fe(III) in clay minerals, thereby modifying mineral structure and redox behavior [9,39]. In marine environments, cyanobacteria such as *Synechococcus* can form mineral–biological aggregates with clay particles, influencing the transport and transformation of metallic elements [35]. Some pathogenic bacteria, such as *E. coli O157:H7*, have also been shown to alter their growth patterns, biofilm formation, and virulence in response to clay associations [36]. The production of EPS by microorganisms constitutes a fundamental mechanism governing clay mineral dynamics [40]. As a representative example, *Pseudomonas* species synthesize EPS biopolymers that adsorb onto mineral surfaces, stabilizing clay–microbe aggregates and promoting mineral dissolution and transformation through processes such as chelation and surface complexation [41].

Fungi also play a significant role in the transformation process of clay minerals. Specific fungal taxa (such as *Aspergillus* species) accelerate the dissolution of clay structures by secreting organic acids, and thereby releasing key structural elements (such as aluminum and silicon) and facilitating mineral breakdown [3]. Concurrently, the filamentous growth morphology of fungi (such as filamentous fungi and mycorrhizal fungi) can promote the aggregation of clay particles and create spatially heterogeneous microenvironments that further influence mineral transformation processes [33,34].

Although archaea are generally more restricted in their distribution, they exert a disproportionately important influence in extreme habitats. Notably, halophilic and thermophilic archaea interact with specific clay minerals (such as montmorillonite and silicate minerals) under conditions of high salinity, temperature, or pH stress, thereby modulating the chemical state and geochemical behavior of the mineral surfaces [3].

**Table 2:** Representative microorganisms involved in clay mineral interactions and their functional roles.

Microbial Group	Representative Genera/Species	Key Functions/Environments	References
Bacteria	*Shewanella*, *Geobacter*, *Pseudomonas*, *Bacillus*, *E. coli*, *Synechococcus*	Iron reduction, biofilm formation, metal cycling, soil/rhizosphere	[9,34,35,36,38,39]
Fungi	*Aspergillus* spp., *Glomeromycota*	Biobeneficiation, soil aggregation, nutrient cycling	[33,34,42]
Archaea	Halophilic archaea, Thermophilic archaea	Extreme/anoxic environments	[3]

## 3. Mechanisms of Clay Mineral–Microorganism Interactions

### 3.1. Physical and Biochemical Regulatory Mechanisms

**Figure 4:**
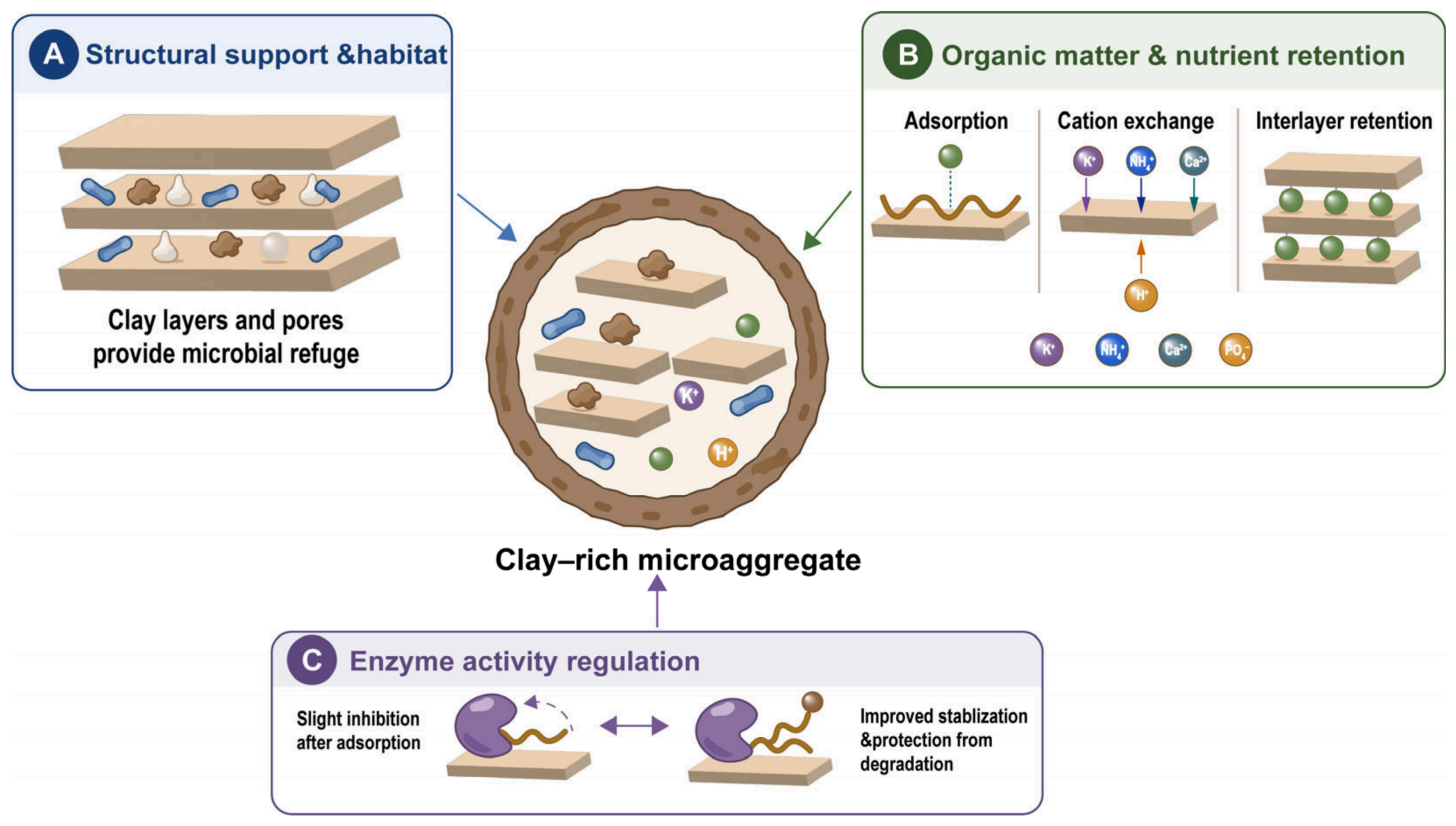
Protective and regulatory roles of clay minerals in microbial systems: (**A**) structural support and habitat provision; (**B**) organic matter and nutrient retention; (**C**) enzyme activity regulation.

Physical interactions between clay minerals and microorganisms involve multiple regulatory processes, including habitat formation, organic matter and nutrient retention, and modulation of extracellular enzyme activities. These major functions are summarized in Figure 4.

#### 3.1.1. Structural Support and Habitat Provision

Clay minerals serve as a fundamental component of soil ecosystems, providing structural integrity and creating protective micro-environments that regulate microbial colonization and stabilize organic matter (Figure 4A). These roles are critical in governing nutrient cycling, sustaining biological activity, and maintaining the long-term ecological functioning of soils. Micro-aggregates enriched with clay minerals function as essential ecological units, distinguished by their unique pore structures and mineral characteristics. With porosity typically ranging from 15% to 35%, their internal pore networks not only offer physical refuge and habitats for microorganisms but also control the movement of water and solutes. These microstructures further act as buffers against environmental stressors, including drought and extreme pH, thereby improving microbial resilience [43].

From a structural perspective, layered silicate minerals—particularly montmorillonite and illite—contribute to the physical framework of micro-aggregates through interlayer spaces and sheet stacking, which promote particle association, pore formation, and mechanical stability. These features enhance aggregate integrity and resistance to external disturbances.

From a habitat perspective, the same mineral-controlled pore architecture creates heterogeneous microenvironments that regulate microbial activity. The hydration and swelling behavior of clay minerals, particularly montmorillonite, can modify pore structure and permeability, while changes in hydration state further regulate the mobility and diffusion of water and interlayer ions [44,45]. These changes alter local water and solute transport conditions and may consequently influence nutrient availability within mineral-associated microbial habitats.

Recent studies further demonstrate that clay mineral-controlled pore architecture and water–solute dynamics play a decisive role in shaping micro-habitat heterogeneity, regulating microbial distribution, and mediating nutrient transport within micro-aggregates [46,47].

Additionally, organo-mineral complexes formed through interactions between clay surfaces and organic matter contribute to the stabilization of soil aggregates and regulate the accessibility and persistence of organic substrates in microbial habitats [32].

#### 3.1.2. Organic Matter and Nutrient Retention

Clay minerals are crucial in retaining organic matter and nutrients in soil systems, mainly through surface adsorption, cation exchange, and interlayer entrapment mechanisms (Figure 4B). By sequestering organic compounds, they establish a physical and chemical barrier that limits the bioavailability of organic matter and slows carbon mineralization, thereby improving the stability of the soil carbon pool [48]. Recent experimental evidence further supports this mechanism. For instance, Sridevi et al. investigated the effects of long-term fertilization on SOC stocks in an Inceptisol containing abundant 2:1 clay minerals, including montmorillonite, vermiculite, and illite. Their results showed that balanced fertilization combined with farmyard manure (100% NPK + FYM) significantly increased SOC stocks and carbon sequestration compared with unfertilized soils, highlighting the role of clay mineral–organic matter interactions in soil carbon stabilization [17,49]. Similarly, Lieberman et al. emphasized that phosphorus can be strongly enriched in mineral-associated organic matter and may act as a dominant control on soil organic matter (SOM) persistence, suggesting that nutrient–mineral interactions are central to long-term carbon stabilization [50].

A key attribute of clay minerals is their cation exchange capacity (CEC), which enables the retention and exchange of nutrient cations such as K^+^, NH_4_^+^, and Ca^2+^. Phosphorus retention, in contrast, is primarily governed by surface complexation, interactions with Fe/Al-bearing phases, and precipitation reactions. This capacity helps minimize nutrient leaching while sustaining microbial metabolic processes [51,52,53]. Recent studies have provided more specific mechanistic evidence. Tang et al. showed that phosphate retention depends strongly on mineralogical composition and pH, with phosphate being retained through inner-sphere complexation on iron oxides under acidic to neutral conditions, whereas exchangeable Ca^2+^ released from layered silicate minerals contributed to hydroxyapatite precipitation under alkaline conditions [54]. Ge et al. further reviewed that phosphorus at soil mineral–organic carbon interfaces is dynamically regulated by adsorption, precipitation, desorption, and dissolution processes, highlighting the close coupling between mineral surfaces, organic carbon, and nutrient availability [55].

Under environmental stressors such as drought or salinization, these nutrient retention properties help maintain a continuous supply of microbial substrates, alleviating nutrient limitation and supporting ecosystem functionality. Recent field evidence shows that nutrient addition can alter exchangeable cation distributions and improve the thermal stability of soil organic matter, especially within micro-aggregates, indicating that exchangeable K^+^, Na^+^, Ca^2+^, and Mg^2+^ are directly involved in regulating aggregate stability and SOM persistence [56]. Moreover, studies on clay-rich soils indicate that CEC and specific surface area strongly influence microbially induced mineral precipitation, suggesting that clay mineral properties can regulate microbial processes by controlling ion availability and reaction sites [57].

Furthermore, the selective ion affinity of certain clay minerals improves nutrient retention and availability in soil systems. Illite and other 2:1 phyllosilicates can retain NH_4_^+^ through adsorption and fixation at surface, edge, and interlayer-associated sites, thereby reducing nitrogen loss and contributing to inorganic nitrogen preservation in soils [58,59]. Similarly, montmorillonite and related layered silicates can regulate Ca^2+^- and P-associated reactions, affecting nutrient availability and microbial processes involved in phosphate transformation and acquisition. These findings indicate that clay minerals do not merely act as passive nutrient reservoirs; rather, they actively regulate nutrient retention, exchange processes, and microbial accessibility to mineral-associated resources (Figure 4). These functions highlight their important role in soil ecological processes and their potential applications in soil management and remediation.

#### 3.1.3. Enzyme Activity Regulation (Bidirectional Effects)

Clay mineral–microorganism interactions can exert bidirectional effects on enzyme activity through physical protection mechanisms (Figure 4C). The close association between extracellular enzymes and clay mineral surfaces can either inhibit or stabilize enzymatic function.

Adsorption of enzymes onto clay minerals often reduces catalytic efficiency due to conformational changes or blockage of active sites. Studies have shown that strong binding to clay surfaces—due to molecular deformation or hindered substrate access—leads to decreased activity in extracellular enzymes such as acid phosphatases [60]. Electrostatic interactions with negatively charged clay surfaces can distort the enzyme’s native conformation, leading to partial inactivation or reduced reaction rates. Conversely, clay minerals also play a protective role that can significantly prolong the functional lifespan of enzymes in soil. By adsorbing enzymes, clay particles can shield them from proteolytic attack and mitigate damage caused by reactive oxygen species [61] and proteases in the soil matrix [62,63].

The interaction between clay minerals and extracellular enzymes exhibits bidirectional effects. Although adsorption onto clay surfaces may reduce enzyme activity by restricting substrate accessibility or altering enzyme conformation, it can also protect enzymes from degradation and enhance their stability [62]. For example, Olagoke et al. demonstrated that mineral adsorption decreased the initial activity of extracellular enzymes but improved their resistance to degradation. This balance between enzymatic inhibition and stabilization allows clay minerals to regulate enzyme persistence and organic matter turnover in soil ecosystems [63].

### 3.2. Chemical Regulatory Mechanisms

**Figure 5:**
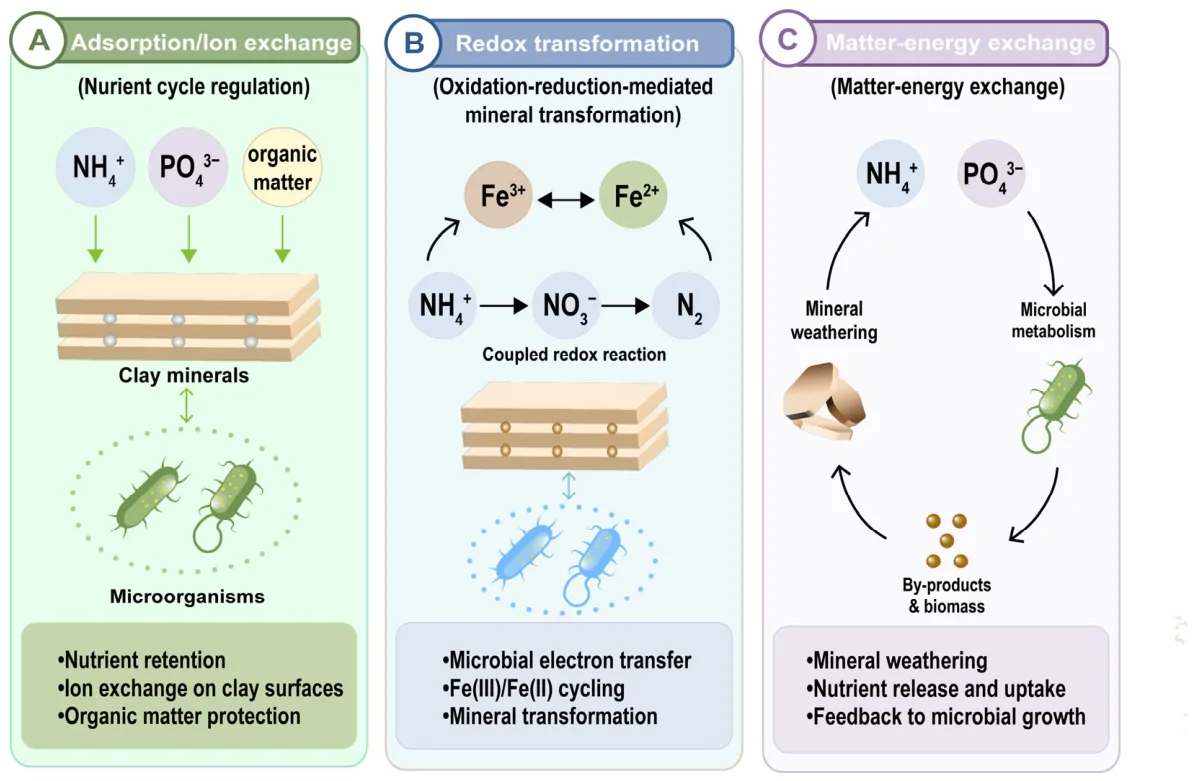
Chemical regulatory mechanisms of clay mineral–microorganism interactions: (**A**) adsorption and ion exchange; (**B**) redox transformation; (**C**) matter–energy exchange.

Clay minerals and microorganisms regulate elemental cycling through three major processes: mineral surface adsorption, microbially mediated transformation, and redox-driven mineral reactions (Figure 5). These processes collectively influence carbon stabilization, nitrogen conversion, phosphorus mobilization, and sulfur transformation.

#### 3.2.1. Nutrient Cycle Regulation

**Figure 6:**
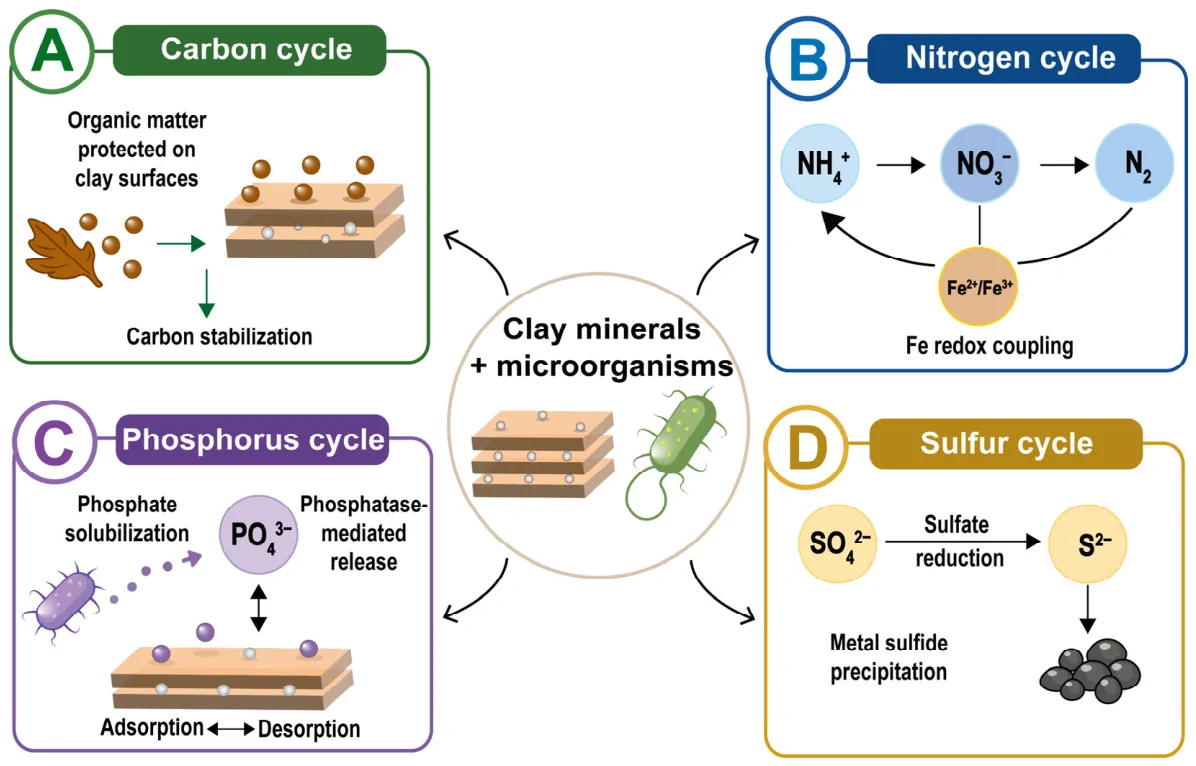
Representative clay mineral–microorganism processes involved in biogeochemical cycling: (**A**) carbon cycle; (**B**) nitrogen cycle; (**C**) phosphorus cycle; (**D**) sulfur cycle.

Nutrient cycling represents one of the most important chemical regulatory functions of clay mineral–microorganism interactions. Clay minerals regulate the retention, transformation, and availability of essential elements through adsorption, ion exchange, and mineral–microbe interactions, whereas microorganisms drive element conversion through metabolic activities and enzymatic processes. Because each elemental cycle involves numerous interconnected reactions, Figure 6 highlights only representative clay mineral–microorganism processes relevant to carbon, nitrogen, phosphorus, and sulfur cycling.

(1) Carbon cycle regulation

The preservation of organic matter is a key process controlling carbon dynamics in terrestrial ecosystems, and clay minerals play an important role by regulating the stabilization and accessibility of soil organic carbon. Organic–mineral associations formed through interactions between clay surfaces and organic compounds can protect organic matter from microbial decomposition by promoting physical occlusion, adsorption, and chemical stabilization. These processes are strongly influenced by clay mineral properties, including surface area, charge characteristics, and mineral structure, which determine the persistence and availability of mineral-associated organic carbon [17,32].

Clay minerals do not simply act as passive carbon reservoirs; rather, they regulate carbon cycling by controlling the balance between organic matter protection and microbial utilization (Figure 6A). Organic compounds adsorbed onto clay surfaces may become less accessible to microorganisms, whereas microbial activities can modify mineral surfaces through extracellular enzyme secretion, metabolite production, and mineral transformation, thereby influencing the turnover of mineral-associated organic matter. Therefore, clay–microorganism interactions represent an important mechanism linking microbial carbon processing with long-term carbon stabilization in soils [17].

Moreover, the interaction between clay minerals and microorganisms determines whether mineral-associated organic carbon is preserved or released. Microbial colonization of clay surfaces, extracellular polymeric substance production, and enzymatic degradation processes collectively regulate organic matter accessibility and decomposition rates. Understanding these interfacial processes provides important insights into carbon stabilization mechanisms and supports the development of clay-based strategies for improving soil carbon retention and ecosystem sustainability.

(2) Nitrogen cycle regulation

Mineral–microbe interactions play a central role in regulating the nitrogen cycle, particularly nitrification, denitrification, anaerobic ammonium oxidation, and other redox-coupled nitrogen transformations. A key linkage in these processes is the coupling between the structural iron cycle in clay minerals and nitrogen transformations. This coupling involves redox-driven conversions between NH_4_^+^, NO_3_^−^, and N_2_, with Fe^2+^/Fe^3+^ acting as electron donors or acceptors in these reactions (Figure 6B). For instance, the nitrate-dependent iron oxidation process mediated by microorganisms (Nitrate-Dependent Fe(II) Oxidation, NDFO) involves iron oxidation by iron-oxidizing microorganisms in the clay mineral structure, while simultaneously reducing nitrate. This process can generate nitrogen intermediates and products such as NO_2_^−^, NO, and N_2_O before the formation of N_2_ [64].

Furthermore, under anaerobic conditions, certain autotrophic microorganisms mediate the reduction of Fe(III) to Fe(II), coupled with the oxidation of ammonium NH_4_^+^ to N_2_, NO_2_^−^, or NO_3_^−^ [65]. Denitrifying bacteria such as *Pseudomonas stutzeri* further contribute to this network by reducing nitrate to gaseous nitrogen forms (e.g., N_2_) through enzymatically mediated denitrification [11]. Furthermore, NH_4_^+^ retention by clay-rich soil matrices can influence microbial nitrification by regulating substrate availability. Hommes et al. (1998) showed that adsorption of NH_4_^+^ onto soil colloids reduced dissolved ammonium availability and consequently decreased nitrite production by *Nitrosomonas europaea*, highlighting the role of mineral-associated nutrient retention in microbial nitrogen transformation [66].

(3) Phosphorus cycle regulation

Microbial regulation of phosphorus cycling is primarily mediated through biochemical processes that directly alter mineral phosphate solubility. For example, phosphate-solubilizing bacteria have been shown to significantly increase available phosphorus in soils by secreting organic acids that reduce pH and enhance the dissolution of mineral phosphates [67]. Similarly, microbial production of phosphatase enzymes plays a key role in mineralizing organic phosphorus compounds, particularly under phosphorus-limited conditions, thereby increasing bioavailable P pools [68]. These processes—phosphate solubilization and phosphatase-mediated release—collectively drive the mobilization of PO_4_^3−^ from mineral-bound forms, making phosphorus more accessible to biota (Figure 6C).

At the mineral interface, specific interactions between phosphate ions and mineral surfaces further regulate phosphorus dynamics. Phosphate is strongly retained on Fe and Al oxides via inner-sphere complexation, but this retention can be disrupted by microbial metabolites that compete for adsorption sites, leading to enhanced desorption and mobilization [69]. In addition, phosphate retention mechanisms vary with mineral composition and environmental conditions, with adsorption dominating under acidic conditions and precipitation processes becoming more important under alkaline conditions [54].

Furthermore, recent experimental studies have revealed that phosphorus cycling is closely linked to mineral-associated organic matter formation. Phosphorus availability has been shown to directly influence the stabilization of mineral-associated organic carbon, indicating that phosphorus dynamics are tightly coupled with carbon cycling processes [50].

These findings collectively demonstrate that phosphorus cycling in soil systems is governed by a dynamic interplay between microbial metabolism, enzymatic activity, and clay mineral surface reactions. As emphasized by Zhou et al. [70], interactions between soil mineralogy and microorganisms regulate phosphorus availability through changes in phosphorus adsorption, transformation, and mobilization processes [70].

Sulfate-reducing bacteria (SRB) play important roles in sulfur transformation processes within clay-rich environments through their interactions with mineral phases (Figure 6D). By reducing sulfate to sulfide, SRB can promote the transformation of metal-bearing minerals and influence the distribution and stability of associated contaminants. For example, Yan et al. demonstrated that SRB activity in clay-size soil fractions promoted mineral transformation processes and affected the fate of Cd, highlighting the importance of microbial–mineral interactions in contaminant immobilization [71]. These processes are strongly regulated by local redox conditions, pH, and mineral composition, which collectively determine sulfur speciation and the mobility of hazardous elements. Therefore, interactions between SRB and clay-associated mineral phases represent an important pathway linking microbial metabolism with sulfur cycling and environmental remediation. 

#### 3.2.2. Oxidation-Reduction-Mediated Mineral Transformation

Oxidation-reduction processes governing mineral transformation in soils arise from both abiotic mineral redox reactions and microbially mediated electron transfer processes. Redox-active minerals, particularly Fe-bearing minerals, interact with microorganisms through electron-transfer processes, resulting in mineral-phase transformation and associated nutrient cycling (Figure 5B). Among these, microbial metabolism plays a central role in driving redox transformations of mineral phases, thereby regulating nutrient availability and biogeochemical cycling.

From a biotic perspective, diverse microorganisms, including iron-reducing bacteria and fungi, actively catalyze redox reactions by transferring electrons to or from mineral surfaces. For example, Ectomycorrhizal fungi can modify mineral-associated organic matter through extracellular metabolites, some of which exhibit Fe(III)-reducing activity and may alter reactions at iron-mineral surfaces [72]. Similarly, microorganisms can reduce Mn(IV) oxides through stepwise electron-transfer processes, producing soluble Mn intermediates and ultimately Mn(II)-bearing products, thereby altering manganese speciation and mineral transformation [73].

From an abiotic perspective, the redox cycling of mineral phases—particularly iron and manganese oxides—controls the stability and transformation of associated nutrients. For instance, under reducing conditions, Fe(III) minerals are converted to Fe^2+^, which can subsequently react with phosphate to form secondary minerals such as vivianite (Fe_3_(PO_4_)_2_·8H_2_O), thereby regulating phosphorus immobilization and mobility in soils [74]. Variations in redox conditions can also alter the crystallinity, reactivity, and dissolution kinetics of iron minerals, ultimately influencing nutrient stability and bioavailability [75].

Importantly, microbe–mineral interactions are often mediated by extracellular electron transfer (EET) processes. Microorganisms can utilize conductive soil components, such as humic substances, or secrete redox-active compounds, including flavins and quinones, which function as electron shuttles to accelerate mineral reduction. For example, *Geobacter* species have been shown to reduce Fe(III) minerals via electron transfer mediated by humic substances such as fulvic acids, thereby enhancing iron reduction rates and influencing mineral transformation pathways [76].

Collectively, these findings demonstrate that oxidation-reduction-driven mineral transformation is not solely a chemical process but is governed by tightly coupled interactions between microbial metabolism, electron transfer pathways, and mineral redox reactivity.

#### 3.2.3. Matter–Energy Exchange

Clay minerals regulate microbial matter and energy acquisition by controlling the retention, accessibility, and transformation of organic compounds and nutrients at mineral surfaces. Through adsorption, ion exchange, and surface interactions, clay minerals can preserve organic substrates and retain essential nutrients, thereby affecting their availability for microbial utilization. The high cation exchange capacity (CEC) of clay minerals contributes to nutrient retention by regulating the exchange and release of ions such as NH_4_^+^, which influences nutrient availability in mineral-associated microbial habitats [51]. Therefore, clay minerals function not only as physical supports for microorganisms but also as reactive interfaces that regulate resource exchange between minerals and microbial communities.

Microorganisms, in turn, modify clay mineral properties through metabolic activities. The production of organic acids, chelating compounds, and redox-active metabolites can promote mineral dissolution, element release, and surface transformation. For example, microbial activity has been shown to induce structural changes and transformation of clay minerals, affecting the release and redistribution of mineral-associated elements [77]. These reciprocal interactions between microbial metabolism and clay mineral transformation establish a dynamic feedback mechanism that regulates nutrient availability and maintains microbial habitats in soil environments [78,79].

### 3.3. Biomineralization and Microbial Regulation of Clay Mineral Transformation

**Figure 7:**
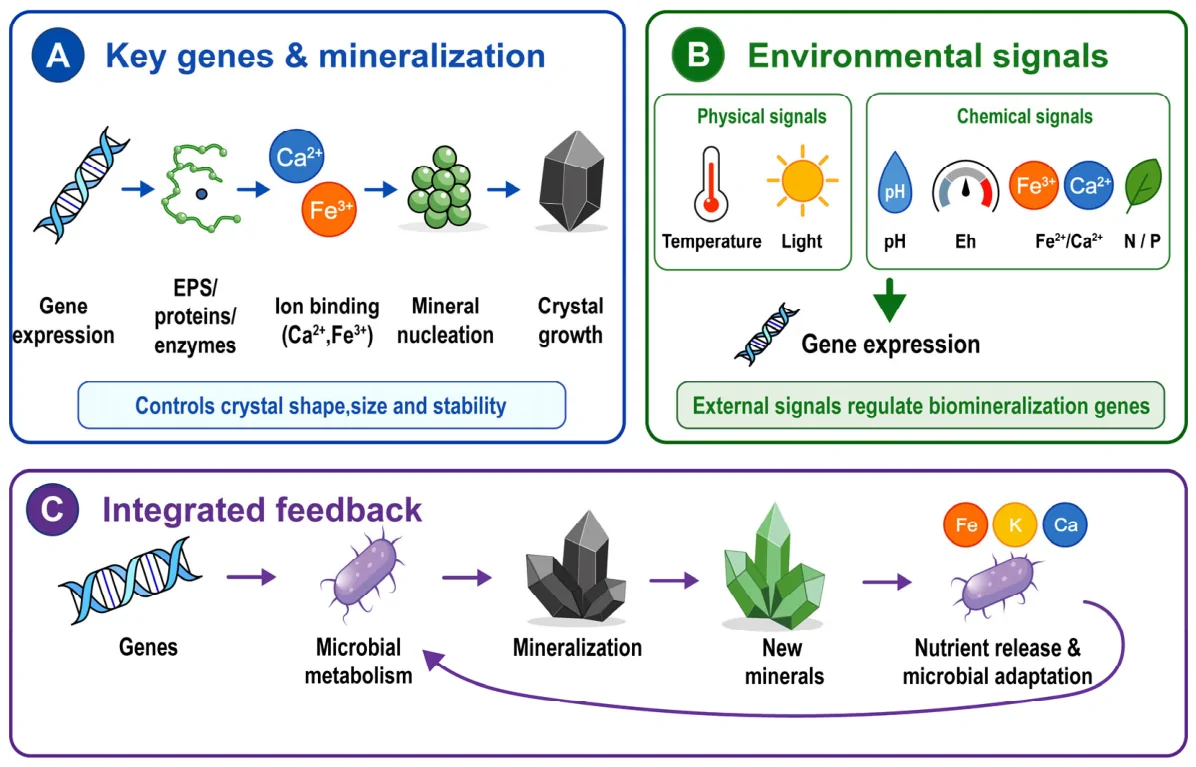
Microorganism-mediated biomineralization processes and mineral–microbe feedback interactions: (**A**) key genes and mineralization; (**B**) environmental signals; (**C**) integrated feedback.

Microorganisms regulate clay mineral transformation through coordinated genetic regulation, environmental responses, and mineral–microorganism feedback processes. These interactions occur at mineral interfaces, where microbial-derived extracellular components, metabolites, and electron transfer processes influence mineral dissolution, secondary mineral formation, and elemental redistribution. Meanwhile, clay mineral properties regulate microbial colonization and metabolic activities by controlling nutrient availability and reactive surface conditions. The major regulatory pathways involved in microbial regulation of clay mineral transformation are summarized in Figure 7.

#### 3.3.1. Key Genes and Mineralization Pathways

Microbial regulation of clay mineral transformation is closely associated with genetic pathways controlling extracellular components, metabolic processes, and mineral–microorganism interactions. Genes involved in extracellular polymeric substance (EPS) production and surface protein expression enable microorganisms to modify mineral interfaces by mediating cell attachment, ion binding, and the formation of reactive microenvironments. EPS contains abundant functional groups, such as hydroxyl and carboxyl groups, which can interact with metal ions and mineral surfaces, thereby influencing mineral nucleation, dissolution, and secondary mineral formation [80,81].

In addition to extracellular components, microbial electron transfer pathways play important roles in regulating redox-sensitive mineral transformation. For example, iron-reducing microorganisms such as *Geobacter* spp. utilize extracellular electron transfer mechanisms to reduce Fe(III)-bearing minerals, resulting in changes in mineral structure and the redistribution of associated elements [82]. These processes demonstrate that microbial genetic regulation can indirectly control mineral transformation by modifying mineral reactivity and elemental mobility.

Furthermore, microbial metabolites, including organic acids and reducing compounds, can alter clay mineral surfaces by promoting mineral dissolution and releasing structurally bound elements. The released elements may participate in secondary mineral formation or become available for microbial utilization, establishing a dynamic feedback between microbial metabolism and clay mineral transformation. Microbial genes, EPS, proteins, and enzymes regulate ion binding, mineral surface interactions, and mineral transformation, thereby influencing elemental cycling and the environmental functions of clay–microorganism systems (Figure 7A).

#### 3.3.2. Environmental Regulation of Clay-Associated Mineral Transformation

Environmental conditions strongly regulate clay-associated mineral transformation by influencing both microbial metabolism and mineral reactivity. Among these factors, pH, redox potential (Eh), dissolved ion concentrations, and nutrient availability are particularly important because they affect mineral dissolution, elemental speciation, and secondary mineral formation. These environmental factors regulate interactions between microorganisms and mineral surfaces, thereby influencing subsequent mineral transformation processes (Figure 7B).

pH is a major control on microbial activity, mineral surface charge, metal solubility, and mineral precipitation. In iron-associated systems, changes in pH can alter microbial Fe oxidation pathways and influence the formation of secondary Fe minerals [83,84]. Redox conditions similarly regulate the transformation of Fe-bearing minerals by controlling the relative abundance and reactivity of Fe^2+^ and Fe^3+^. These redox-driven changes can further affect the mobility and redistribution of mineral-associated elements. In addition, the availability of dissolved ions and nutrients influences microbial metabolism and mineral–microorganism interactions by modifying reaction conditions at mineral surfaces [9,78].

Microbial responses to these environmental conditions can further modify mineral transformation through the production of extracellular polymeric substances, organic acids, and other reactive metabolites. These compounds can promote mineral dissolution, ion complexation, and the formation of secondary mineral phases. For example, microbial extracellular polymers have been shown to influence silicate mineral dissolution and mineral-surface reactions [85]. Therefore, environmental factors do not act independently but jointly regulate microbial activity, mineral reactivity, and the feedback processes that control clay-associated mineral transformation.

#### 3.3.3. Integrated Network of Genes, Metabolism and Mineralization

From the genetic level to mineral formation, microbial physiological metabolism and genetic expression are deeply coupled, forming a complex integrated network.

(1) The transmission path from genes to metabolism

From genetic regulation to mineral transformation, microbial gene expression and metabolic activity are closely linked at clay–microorganism interfaces. Genes involved in extracellular enzyme production, extracellular polymeric substance (EPS) synthesis, electron transfer, and metabolite secretion regulate the production of reactive extracellular components that interact with clay mineral surfaces. These microbial products can promote mineral dissolution, redox reactions, and the redistribution of mineral-associated elements. In turn, changes in mineral surface properties and element availability can influence microbial metabolism and subsequent mineral transformation. Thus, genetic regulation affects clay mineral transformation primarily through its control of microbial metabolic activities and extracellular interactions [9,78].

(2) Ecological effects of the mineralization feedback network

The secondary minerals formed during mineralization processes play a critical role in regulating soil structural and ecological functions. These newly formed mineral phases, including iron (oxyhydr) oxides and clay-associated precipitates, enhance soil structural stability by acting as binding agents that promote aggregate formation and protect organic matter from decomposition [86]. In parallel, these mineral products serve as dynamic nutrient reservoirs by adsorbing and retaining essential elements, thereby buffering nutrient fluctuations and sustaining long-term nutrient availability. This integrated feedback loop links genetic regulation, microbial metabolism, and mineral transformation, with newly formed minerals subsequently influencing nutrient release and microbial adaptation (Figure 7C). Recent studies show that mineral weathering-derived nutrients (e.g., Fe, Ca, Si) can stimulate microbial biomass and carbon sequestration, while mineral substrates also shape microbial community composition and functional traits [79]. In turn, microbial metabolites further accelerate mineral transformation, forming a coupled feedback loop [78].

These interactions collectively regulate microbial community adaptation and ecosystem functioning, thereby linking mineral transformation with long-term soil stability [87,88].

## 4. Environmental Remediation and Engineering Applications

### 4.1. Natural Clay Mineral–Microorganism Systems for Heavy-Metal Remediation

**Figure 8:**
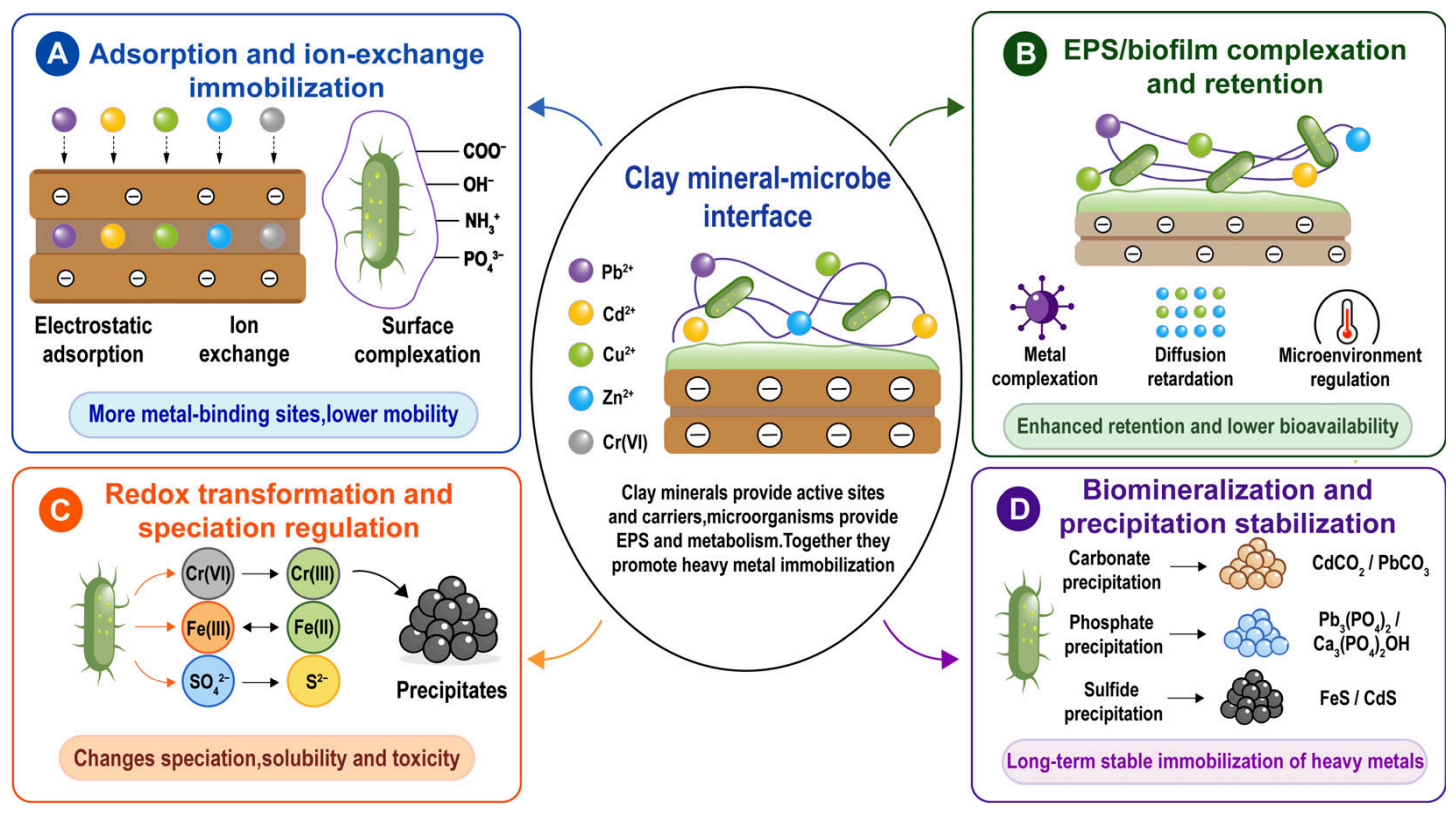
Application of clay mineral–microorganism synergy in heavy-metal remediation: (**A**) adsorption and ion-exchange immobilization; (**B**) EPS/biofilm complexation and retention; (**C**) redox transformation and speciation regulation; (**D**) biomineralization and precipitation stabilization.

Clay mineral–microorganism interactions contribute to heavy-metal immobilization through several interconnected pathways, including adsorption and ion exchange, EPS/biofilm-mediated retention, redox transformation, and biomineralization. In natural soils and sediments, these processes rarely occur independently. Clay mineral surfaces provide adsorption sites, exchangeable cations, and heterogeneous microhabitats for microorganisms, whereas microbial colonization, extracellular polymeric substances (EPS), and metabolic activity can modify mineral surface chemistry and metal speciation. The principal pathways involved in heavy-metal immobilization are summarized in Figure 8. To complement this mechanistic framework, representative quantitative performances of clay mineral–microorganism systems are compared in Table 3.

(1) Adsorption and ion-exchange immobilization

Clay mineral–microorganism systems primarily immobilize heavy metals through adsorption and ion exchange. At the molecular scale, metal retention in clay-rich materials is controlled by multiple coexisting processes, including cation exchange, surface complexation, surface precipitation, and nucleation growth [89]. These mechanisms are consistent with general heavy-metal adsorption pathways, such as electrostatic attraction, ion exchange, complexation, and surface precipitation [90].

In microbial–clay mineral systems, clay minerals provide charged basal surfaces, edge sites, interlayer exchange sites, and surface hydroxyl groups, whereas microbial cells and EPS contribute additional metal-binding functional groups. This interfacial coupling can measurably enhance metal retention. For example, at Cd concentrations of 16–128 mg L^−1^, a montmorillonite–*Chryseobacterium* sp. WAL2 system retained 67.6–82.1% of Cd, compared with 60.6–67.2% for montmorillonite alone and 2.57–5.94% for free bacterial cells [91]. The combined adsorption exceeded the summed contributions of the individual components, indicating a synergistic mineral–microbe effect. Montmorillonite also regulated genes associated with ion transport, efflux pumps, oxidative stress, and intracellular Cd handling, linking interfacial retention with microbial adaptation to Cd stress. These processes contribute jointly to heavy-metal immobilization at the mineral–microbe interface (Figure 8A).

Microbial weathering of montmorillonite enhanced Cd(II) immobilization by exposing additional reactive sites and introducing microbial-derived functional groups. The maximum Cd(II) adsorption capacity increased from 29.65 mg/g for raw montmorillonite to 41.85–52.43 mg/g for bacteria–montmorillonite complexes, corresponding to an increase of up to 104.45% after normalization to the montmorillonite fraction [92]. EPS from *Microcystis aeruginosa* enhanced Cu(II) adsorption onto kaolinite mainly through complexation and the creation of additional adsorption sites at the kaolinite–EPS interface. At an EPS concentration of 0.3 g/L, Cu(II) adsorption reached 353.01% of that observed for pure kaolinite [93]. Mineral-associated EPS from *Bacillus subtilis* also increased heavy-metal sorption by bentonite; Pb^2+^ sorption increased by 20%, while its initial sorption rate increased by 151% compared with uncoated bentonite [94]. These results show that mineral-bound EPS can increase both the number of available binding sites and the kinetics of metal retention at clay surfaces.

(2) EPS/biofilm-mediated complexation and retention

EPS and biofilms serve as key biological interfaces for heavy-metal retention. EPS contains polysaccharides, proteins, extracellular DNA, lipids, and other biopolymers that promote microbial adhesion, biofilm stability, mineral aggregation, and metal complexation. Once microorganisms colonize clay surfaces, the EPS-rich biofilm matrix can trap metal ions, reduce diffusion, and create local gradients of pH, redox potential, organic ligands, and competing ions, thereby regulating metal speciation and bioavailability.

Clay minerals can also facilitate microbial attachment and biofilm development by providing reactive surfaces and protected microhabitats. In *Serratia marcescens* S14, the combined presence of clay minerals and Cd(II) promoted biofilm formation much more strongly than either factor alone. Relative to the control, biofilm biomass increased to 6.25, 3.79, and 11.9 times the control level in the goethite+Cd(II), kaolinite+Cd(II), and montmorillonite+Cd(II) systems, respectively [95]. EPS/biofilm matrices can further enhance metal retention through complexation, diffusion retardation, and microenvironmental regulation at the mineral–microbe interface (Figure 8B).

EPS-producing bacteria can also alter heavy-metal partitioning at the soil–plant interface. Inoculation with *Pseudomonas* sp. H7 and *Agrobacterium* sp. Z22 reduced Cd concentrations in pakchoi tissues by 30.7–81.8% and Pb concentrations by 8.1–57.0% relative to the control, while promoting the formation of CdCO_3_, PbCO_3_, Cd_2_(OH)_2_CO_3_, and 2PbCO_3_·Pb(OH)_2_ in soil microaggregates [96]. Similar effects were observed for *Pseudoalteromonas agarivorans* Hao 2018, which reduced available Pb in contaminated soil by 34.8–38.1% and decreased Pb concentrations in edible pak choi tissues by 14.5–39.2% [97]. EPS can also participate in mineral nucleation and precipitation, providing an additional pathway for the stabilization of heavy metals in mineral-associated forms [98].

(3) Redox transformation and speciation regulation

Redox transformation is important for redox-sensitive metals and metalloids, including Cr, As, U, Hg, Fe, and Mn. Many clay minerals contain structural Fe or are associated with Fe/Mn oxides, which can participate in microbial electron-transfer reactions [99]. Iron-reducing, iron-oxidizing, sulfate-reducing, and other electroactive microorganisms can alter metal valence states, mineral phases, solubility, toxicity, and binding affinity [9]. For example, microbial reduction of Cr(VI) to Cr(III) generally favors immobilization because Cr(III) is less soluble, whereas Fe(III) reduction alters the reactivity and stability of Fe-bearing mineral phases. Sulfate reduction can also generate sulfide, which promotes the precipitation of metals as poorly soluble sulfide phases (Figure 8C).

Sulfate-reducing bacteria interacting with clay-size soil fractions promoted Cd redistribution toward more stable mineral-associated forms. After SRB treatment, exchangeable Cd decreased from 1.56–3.63 to 0.42–1.25 mg/kg, whereas the Fe/Mn oxide-bound fraction increased from 0.13–0.27 to 1.40–3.21 mg/kg [71]. In estuarine sediments, enhanced sulfate reduction promoted FeS formation and stabilized heavy metals in the solid phase. In the *Bacillus coagulans*–olivine treatment, approximately 50% of Cd in the −35 cm sediment layer occurred in the residual fraction, while FeS accounted for about 75% of solid-phase sulfur [100].

(4) Biomineralization and precipitation stabilization

Biomineralization provides a relatively stable pathway for long-term heavy-metal immobilization. Microorganisms can induce the formation of carbonate, phosphate, sulfide, Fe, or Mn minerals, while clay minerals enrich metal ions, provide nucleation surfaces, and stabilize newly formed biominerals within biofilms or soil aggregates. Heavy metals may then be immobilized through co-precipitation, lattice substitution, surface adsorption, or physical encapsulation. In microbially induced carbonate precipitation (MICP) systems, ureolytic microorganisms hydrolyze urea to generate carbonate ions and increase local alkalinity, promoting CaCO_3_ precipitation and stabilizing Cd, Pb, Cu, Zn, and Ni. Recent reviews indicate that bacterial cells and EPS provide nucleation sites, and that MICP stabilizes heavy metals through biosorption, carbonate precipitation, and co-precipitation [101,102]. Beyond carbonate precipitation, phosphate and sulfide precipitation provide additional biomineralization pathways for heavy-metal immobilization (Figure 8D) [103].

Experimental studies provide direct evidence for these biomineralization pathways. A landfill leachate-derived ureolytic bacterial consortium achieved a Cd^2+^ removal efficiency of 99.10% and a Ni^2+^ removal efficiency of approximately 76–78% under optimized MICP conditions, while promoting stable calcite formation [104]. Sulfate-reducing bacteria also showed high mineralization efficiencies for Cd^2+^ and Pb^2+^; *Desulfobulbus propionicus* mineralized 98.97% of Cd^2+^ at an initial concentration of 30 mg/L, whereas both *D*. *propionicus* and *Desulfovibrio desulfuricans* achieved approximately 80% Pb^2+^ immobilization at initial concentrations below 50 mg/L [105]. In a *Chlorella sorokiniana*–montmorillonite system, montmorillonite buffered pH fluctuations during Pb(II) biomineralization and provided surfaces for Pb-bearing mineral formation, narrowing the pH range from 5.07–6.73 for *Chlorella* alone to 5.23–6.27 and 5.49–6.25 for the 1:1 and 1:5 composites, respectively [106].

In practical remediation, these mechanisms often operate simultaneously rather than independently, with clay–microbe interfaces coupling adsorption, biofilm retention, redox transformation, and biomineralization to enhance heavy-metal stabilization.

**Table 3:** Quantitative performance of representative clay mineral–microorganism systems in heavy-metal remediation.

Clay Mineral–Microorganism System	Metal	Key Quantitative Result	Reference
Montmorillonite–*Chryseobacterium* sp. WAL2	Cd	Cd retention: 67.6–82.1%; Mt alone: 60.6–67.2%; cells alone: 2.57–5.94%	[91]
Montmorillonite–*Paenibacillus polymyxa*/*Bacillus circulans*	Cd	Adsorption capacity increased from 29.65 to 41.85–52.43 mg g^−1^	[92]
Kaolinite–EPS from *Microcystis aeruginosa*	Cu	Cu adsorption increased by 253.01% at 0.3 g L^−1^ EPS	[93]
Bentonite–*Bacillus subtilis* EPS	Pb	Pb sorption increased by 20%; initial sorption rate increased by 151%	[94]
Clay minerals–*Serratia marcescens* S14	Cd	Biofilm biomass increased to 3.79–11.9 times the control	[95]
Clay-size fractions–sulfate-reducing bacteria	Cd	Exchangeable Cd: 1.56–3.63 → 0.42–1.25 mg kg^−1^; Fe/Mn oxide-bound Cd: 0.13–0.27 → 1.40–3.21 mg kg^−1^	[71]
Montmorillonite–*Chlorella sorokiniana* FK	Pb	Pb(II) concentration continuously decreased from an initial 150 mg L^−1^, while PbCO_3_, Pb_3_O(CO_3_)_2_H_2_O, and Pb_3_(PO_4_)_2_ were formed during biomineralization.	[106]

### 4.2. Natural Clay Mineral–Microorganism Systems for Organic-Pollutant Remediation

**Figure 9:**
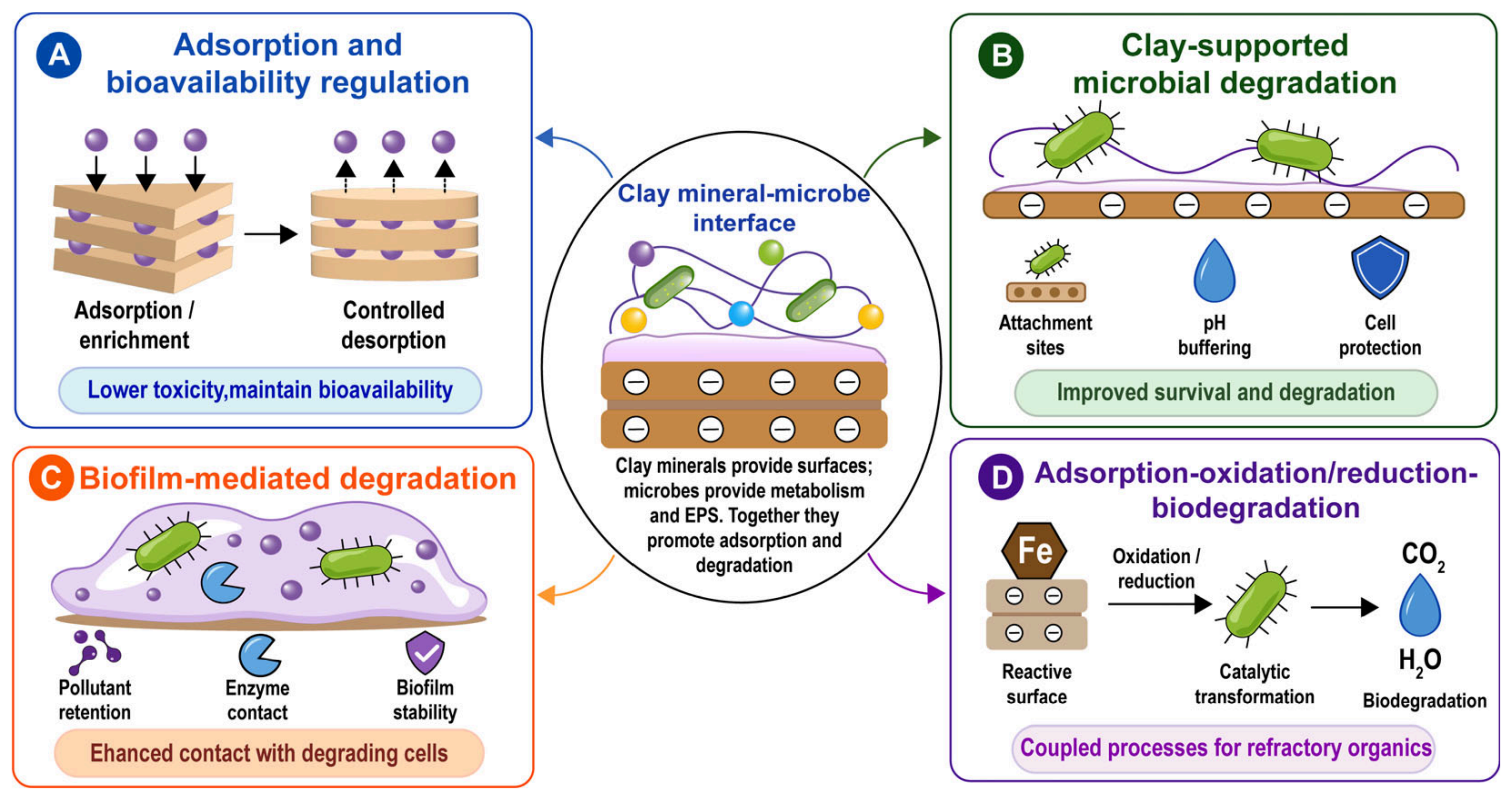
Clay mineral–microorganism synergistic pathways for organic-pollutant remediation: (**A**) adsorption and bioavailability regulation; (**B**) clay-supported microbial degradation; (**C**) biofilm-mediated degradation; (**D**) adsorption–oxidation/reduction–biodegradation.

Clay minerals can influence organic-pollutant remediation by regulating pollutant adsorption, bioavailability, microbial attachment, and biodegradation. In natural clay–microbe systems, these effects are mainly expressed through adsorption-mediated bioavailability regulation, clay-supported microbial degradation, and biofilm-mediated transformation. Clay surfaces can therefore function not only as pollutant sorbents, but also as reactive interfaces that modify microbial colonization and pollutant accessibility (Figure 9A–C).

(1) Adsorption-mediated bioavailability regulation

Clay minerals can adsorb organic pollutants, including antibiotics, dyes, pesticides, phenols, PAHs, and chlorinated organic compounds, through electrostatic attraction, cation exchange, hydrogen bonding, π–π interactions, surface complexation, and pore filling. In microbial systems, adsorption may have dual effects on pollutant transformation [107,108,109]. On the one hand, it can reduce pollutant mobility and acute toxicity; on the other hand, overly strong adsorption may diminish pollutant bioavailability and limit microbial degradation [12]. Therefore, the function of clay minerals is not limited to pollutant removal but also involves regulating pollutant accessibility at the mineral–microbe interface [32]. Adsorption–desorption equilibrium at clay surfaces influences pollutant availability and determines whether adsorbed compounds can be further transformed by microorganisms (Figure 9A).

For example, Sun et al. [110] investigated norfloxacin adsorption by montmorillonite and kaolinite under different aquatic conditions. Montmorillonite showed substantially higher adsorption capacity than kaolinite, with adsorption capacities of 2.880, 2.208, 0.433, and 0.067 mg g^−1^ in simulated freshwater, actual freshwater, simulated seawater, and actual seawater, respectively, whereas kaolinite exhibited values of 0.199, 0.120, 0.094, and 0.010 mg g^−1^ under the corresponding conditions. The adsorption was mainly attributed to electrostatic interactions, cation exchange, and cation bonding/bridging [110]. However, the authors also observed that montmorillonite-bound norfloxacin decreased from 2.880 to 0.300 mg g^−1^ under simulated estuarine conditions, indicating that environmental changes may trigger desorption and potential secondary transport of adsorbed antibiotics. Therefore, adsorption-based immobilization should be coupled with microbial or chemical degradation processes to achieve long-term pollutant removal.

(2) Clay-supported microbial degradation of organic pollutants

Clay minerals can serve as microbial carriers by providing attachment sites and favorable microenvironments for pollutant degradation (Figure 9B). For example, natural kaolin clay was used to immobilize microorganisms for phenol degradation in real industrial wastewater. Compared with free microorganisms, phenol removal increased from 68% to 98% at 50 mg L^−1^ and from 38% to 80% at 300 mg L^−1^ [111]. These results demonstrate that kaolin can enhance microbial degradation of phenol by acting as a carrier rather than merely as an adsorbent. More recently, Peng et al. immobilized *Novosphingobium* sp. RL4 using an attapulgite–sodium alginate carrier for phoxim degradation. The immobilized system degraded 87.70% of 20 mg L^−1^ phoxim within 72 h and retained more than 80% degradation efficiency after five reuse cycles [112].

Another study showed that the atrazine-degrading strain *Arthrobacter* sp. DNS10 cultured with montmorillonite, kaolinite, and goethite showed different responses in growth and functional gene expression. The relative expression of the atrazine-degrading gene trzN reached 0.94-, 0.27-, and 0.20-fold in montmorillonite, kaolinite, and goethite systems, respectively, indicating that mineral type strongly influences microbial degradation potential [113]. Ruan et al. further demonstrated that *Sphingomonas* sp. GY2B interacted with stearic acid-modified montmorillonite during phenanthrene biodegradation. Phenanthrene degradation reached 98% within 2 d, while bacterial activity simultaneously altered the mineral surface and promoted elemental dissolution [15].

(3) Biofilm-mediated degradation on mineral surfaces

Biofilm formation provides an important connection between adsorption and biodegradation. Once microorganisms colonize clay mineral surfaces, EPS-rich biofilms can retain pollutants near microbial cells, enhance cell–pollutant contact, and protect microorganisms against environmental stress. The biofilm matrix also regulates pollutant diffusion and creates favorable microenvironments for enzymatic transformation, thereby promoting organic pollutant degradation (Figure 9C).

Direct evidence for clay-associated biofilm enhancement has been demonstrated in a montmorillonite–*Pseudomonas* sp. Z1–methyl parathion system. The attachment of degrading bacteria onto montmorillonite surfaces enhanced microbial metabolic activity and accelerated methyl parathion degradation. With 20 g L^−1^ montmorillonite, 98.6% of methyl parathion was removed within 10 h, whereas abiotic controls showed less than 1% loss, indicating that mineral-associated microbial activity rather than adsorption alone dominated pollutant removal [114]. These results demonstrate that clay surfaces can function as microbial habitats that enhance biofilm-associated degradation of organic pollutants. Representative quantitative results for organic-pollutant remediation in clay-associated systems are summarized in Table 4.

### 4.3. Engineered and Modified Clay-Based Remediation Systems

Unlike natural clay mineral–microorganism systems, engineered clay-based materials have been developed by modifying clay structures or introducing additional functional components to enhance pollutant removal. These engineered systems combine the intrinsic adsorption ability of clay minerals with additional functions, including catalytic oxidation, redox activity, and enhanced surface reactivity. Such strategies expand the application potential of clay-based materials for the remediation of complex organic and inorganic contaminants.

(1) Clay-based composite materials for enhanced adsorption and immobilization

For example, Hou et al. developed sepiolite-modified biochar for the remediation of Pb, Cd, and Cr co-contaminated soil. Compared with untreated soil, DTPA-extractable Pb decreased by 12.39% and 13.55% after treatment with MBC500 and HBC500, respectively, indicating a reduction in the bioavailable Pb fraction in the treated soil [115]. These results suggest that clay incorporation can reduce Pb availability in biochar-amended soil.

For organic pollutants, engineered clay adsorbents and clay-based nanomaterials have been developed due to their adjustable pore structures, high surface reactivity, and tunable functional groups. Such modifications improve the adsorption of antibiotics, dyes, and other persistent organic contaminants [116]. However, excessive adsorption may result in pollutant accumulation on solid surfaces, and subsequent regeneration or degradation strategies are required to achieve sustainable remediation.

(2) Clay-supported catalytic oxidation systems

Beyond adsorption enhancement, modified clay materials can act as catalytic platforms for oxidative degradation of refractory organic pollutants. The introduction of redox-active metals, especially Fe species, provides additional catalytic sites and promotes electron-transfer processes during oxidation reactions.

Boukhemkhem et al. prepared Fe-exchanged bentonite and kaolinite catalysts through ion exchange and calcination, and applied them in heterogeneous Fenton degradation of neonicotinoid pesticides including nitenpyram and acetamiprid [117]. Clay minerals can also promote pollutant oxidation through adsorption-assisted catalytic ozonation. Foka Wembe et al. investigated clay-catalyzed ozonation of atrazine, bisphenol A, diazinon, and diclofenac, demonstrating that clay minerals acted as both adsorbents and catalytic supports during oxidation. The adsorption behavior followed the Langmuir model, and clay composition and surface properties affected pollutant retention and oxidative transformation pathways [118]. These findings indicate that engineered clay materials can enhance remediation by coupling pollutant enrichment with catalytic oxidation.

Although engineered clay-based materials exhibit enhanced remediation performance, most studies remain focused on laboratory-scale applications. Future research should further address catalyst regeneration, metal leaching, transformation product toxicity, and the integration of engineered clay materials with biological remediation approaches.

## 5. Conclusions and Future Perspectives

### 5.1. Conclusions

Microbial–clay mineral interactions represent key interfacial processes that couple microbial metabolism, mineral transformation, biogeochemical cycling, and environmental remediation. Clay minerals provide microorganisms with protective habitats, essential nutrients, reactive surfaces, and adsorption sites, while microorganisms reciprocally modify clay minerals through EPS secretion, organic acid production, redox reactions, and biomineralization. These reciprocal interactions form a dynamic mineral–microbe interface that regulates pollutant migration, transformation, and stabilization.

In remediation applications, clay mineral–microorganism systems have demonstrated considerable potential for both heavy-metal immobilization and organic-pollutant degradation. For heavy metals, adsorption, ion exchange, EPS-mediated complexation, redox transformation, and biomineralization jointly reduce metal mobility and bioavailability. For organic pollutants, clay minerals can enrich contaminants, support microbial colonization, and enhance biodegradation, thereby coupling adsorption with biological transformation. Despite these advances, several challenges remain. The molecular mechanisms and multi-scale coupling processes at clay–microbe interfaces are still insufficiently understood. Most existing studies have been conducted under simplified laboratory conditions, whereas field environments involve complex variations in pH, redox potential, salinity, dissolved organic matter, nutrients, and microbial communities. In addition, the long-term stability, secondary pollution risk, and ecological safety of these systems require further evaluation.

### 5.2. Future Perspectives

Future research should integrate advanced spectroscopic techniques, high-resolution microscopy, multi-omics approaches (e.g., metagenomics, transcriptomics, proteomics), isotope tracing, and molecular dynamics simulations to elucidate interfacial mechanisms at the molecular level. More attention should be directed toward emerging contaminants (such as microplastics and per- and polyfluoroalkyl substances), mixed pollution systems, and the scalability of laboratory findings to field-scale applications. The development of stable, efficient, and environmentally benign clay mineral–microorganism composites will offer a promising nature-based strategy for sustainable pollution remediation and ecosystem restoration.

In summary, a deeper understanding of microbial–clay mineral interactions will not only advance fundamental knowledge of Earth surface processes but also inform the rational design of next-generation bioremediation technologies, contributing to global efforts in pollution control and environmental sustainability.

## Figures and Tables

**Table 4:** Quantitative performance of representative clay mineral–microorganism systems in organic-pollutant remediation.

Clay Mineral–Microorganism System	Pollutant	Key Quantitative Result	Reference
Kaolin-immobilized microorganisms	Phenol	Phenol removal increased from 68% to 98% at 50 mg L^−1^ and from 38% to 80% at 300 mg L^−1^	[111]
Montmorillonite/kaolinite–*Arthrobacter* sp. DNS10	Atrazine	Relative trzN expression: 0.94-fold (montmorillonite) and 0.27-fold (kaolinite)	[113]
Montmorillonite–*Pseudomonas* sp. Z1	Methyl parathion	Methyl parathion removal reached 98.6% within 10 h; abiotic loss was <1%	[114]
Attapulgite–sodium alginate–*Novosphingobium* sp. RL4	Phoxim	Phoxim degradation reached 87.70% within 72 h and remained >80% after five reuse cycles	[112]
Stearic acid-modified montmorillonite–*Sphingomonas* sp. GY2B	Phenanthrene	Phenanthrene degradation reached 98% within 2 d	[15]

## Data Availability

No new data were created or analyzed in this study. Data sharing is not applicable to this article.

## Supplementary Materials

The following supporting information can be downloaded at: https://www.mdpi.com/article/10.3390/toxics14100900/s1, Table S1: Search strategies used for the bibliometric analysis in Figure 1.
