# Supplementary material for: Microbial–Clay Mineral Interactions: Mechanisms and Applications in Environmental Remediation
Source: Toxics. 2026 Oct 11;14(10):900. doi: 10.3390/toxics14100900 (PMC13709464; doi:10.3390/toxics14100900)
Supplement: Supplementary file 1 [file toxics-14-00900-s001.zip › toxics-4553497-supplementary.pdf]

Table S1. Search strategies used for the bibliometric analysis in Figure 1

Dataset	Figure	Complete Web of Science search strategy
Clay mineral–microorganism interactions	Fig. 1A	TS=(<br>(<br>"clay mineral*" OR clay OR montmorillonite OR kaolinite OR illite OR<br>bentonite OR smectite OR vermiculite OR chlorite<br>)<br>AND<br>(<br>microb* OR microorganism* OR bacter* OR fungi OR fungal OR archae* OR<br>algae OR biofilm OR EPS<br>)<br>AND<br>(<br>interaction* OR association* OR interface* OR adhesion OR colonization OR<br>transformation OR weathering<br>)<br>)
Mineral-assisted bioremediation	Fig. 1A	TS = (<br>(<br>mineral* OR "clay mineral*" OR clay OR montmorillonite OR kaolinite OR illite OR<br>bentonite OR smectite OR vermiculite OR chlorite OR goethite OR ferrihydrite OR hematite OR magnetite<br>)<br>AND<br>(<br>microb* OR microorganism* OR bacter* OR fungi OR fungal OR archae* OR algae OR biofilm OR EPS<br>)<br>AND

		(<br>bioremediation OR biodegradation OR biotransformation OR bioaugmentation OR<br>"microbial remediation" OR "biological remediation" OR detoxification OR immobilization OR<br>"pollutant removal" OR "contaminant removal" OR "environmental remediation"<br>)<br>)
Heavy metals	Fig. 1B	TS=(<br>(<br>mineral* OR "clay mineral*" OR clay OR montmorillonite OR kaolinite OR illite OR<br>bentonite OR smectite OR vermiculite OR chlorite OR goethite OR ferrihydrite OR hematite OR magnetite<br>)<br>AND<br>(<br>microb* OR microorganism* OR bacter* OR fungi OR fungal OR biofilm OR<br>"extracellular polymeric substance*" OR EPS<br>)<br>AND<br>(<br>bioremediation OR remediation OR immobilization OR stabilization OR detoxification OR<br>biosorption OR bioaccumulation OR biomineralization OR biotransformation<br>)<br>AND<br>(

		"heavy metal*" OR "toxic metal*" OR cadmium OR lead OR chromium OR arsenic OR<br>mercury OR copper OR zinc OR nickel OR<br>"Cd(II)" OR "Pb(II)" OR "Cr(VI)" OR "As(III)" OR "As(V)" OR "Cu(II)" OR "Zn(II)" OR "Ni(II)"<br>)<br>)
Hydrophobic organic contaminants (HOCs)	Fig. 1B	TS=(<br>(<br>mineral* OR "clay mineral*" OR clay OR montmorillonite OR kaolinite OR illite OR<br>bentonite OR smectite OR vermiculite OR chlorite OR goethite OR ferrihydrite OR<br>hematite OR magnetite<br>)<br>AND<br>(<br>microb* OR microorganism* OR bacter* OR fungi OR fungal OR biofilm OR<br>"extracellular polymeric substance*" OR EPS<br>)<br>AND<br>(<br>bioremediation OR biodegradation OR biotransformation OR remediation<br>)<br>AND<br>(<br>"hydrophobic organic contaminant*" OR "hydrophobic organic pollutant*" OR<br>"polycyclic aromatic hydrocarbon*" OR PAH* OR<br>phenanthrene OR pyrene OR naphthalene OR anthracene OR

		fluoranthene OR benzoapyrene OR<br>petroleum OR hydrocarbon* OR "petroleum hydrocarbon*" OR "total petroleum hydrocarbon*" OR<br>crude oil OR diesel OR<br>"chlorinated hydrocarbon*" OR trichloroethylene OR tetrachloroethylene OR<br>"polychlorinated biphenyl*" OR PCB*<br>)<br>)
Antibiotics	Fig. 1B	TS=(<br>(<br>mineral* OR "clay mineral*" OR clay OR montmorillonite OR kaolinite OR illite OR<br>bentonite OR smectite OR vermiculite OR chlorite OR goethite OR ferrihydrite OR<br>hematite OR magnetite<br>)<br>AND<br>(<br>microb* OR microorganism* OR bacter* OR fungi OR fungal OR archaea OR archaeal OR<br>algae OR biofilm OR "extracellular polymeric substance*" OR EPS<br>)<br>AND<br>(<br>bioremediation OR biodegradation OR biotransformation OR remediation OR<br>"antibiotic degradation" OR "antibiotic removal"<br>)<br>AND<br>(<br>antibiotic* OR tetracycline OR oxytetracycline OR

chlortetracycline OR doxycycline OR
sulfamethoxazole OR sulfonamide* OR sulfadiazine OR sulfamethazine OR
norfloxacin OR ciprofloxacin OR ofloxacin OR levofloxacin OR enrofloxacin OR
erythromycin OR azithromycin OR chloramphenicol OR amoxicillin
)
)

Database: Web of Science Core Collection;
Search field: Topic (TS);
Document types: Article and Review Article;
Index: SCI-EXPANDED;
Language: English;
Timespan: 1990–2026;
Search date: 20 August 2026.
